# Cardiac Glycosides Activate the Tumor Suppressor and Viral Restriction Factor Promyelocytic Leukemia Protein (PML)

**DOI:** 10.1371/journal.pone.0152692

**Published:** 2016-03-31

**Authors:** Snezana Milutinovic, Susanne Heynen-Genel, Elizabeth Chao, Antimone Dewing, Ricardo Solano, Loribelle Milan, Nikki Barron, Min He, Paul W. Diaz, Shu-ichi Matsuzawa, John C. Reed, Christian A. Hassig

**Affiliations:** 1 Sanford Burnham Prebys Medical Discovery Institute, 10901 N. Torrey Pines Road, La Jolla, CA 92037, United States of America; 2 Bemer USA, LLC, Carlsbad, CA, United States of America; 3 National Cancer Institute (NCI), Bethesda, MD, United States of America; 4 P.William Diaz, Pharmaceutical Consulting, Riverside, CA, United States of America; University of North Carolina at Greensboro, UNITED STATES

## Abstract

Cardiac glycosides (CGs), inhibitors of Na^+^/K^+^-ATPase (NKA), used clinically to treat heart failure, have garnered recent attention as potential anti-cancer and anti-viral agents. A high-throughput phenotypic screen designed to identify modulators of promyelocytic leukemia protein (PML) nuclear body (NB) formation revealed the CG gitoxigenin as a potent activator of PML. We demonstrate that multiple structurally distinct CGs activate the formation of PML NBs and induce PML protein SUMOylation in an NKA-dependent fashion. CG effects on PML occur at the post-transcriptional level, mechanistically distinct from previously described PML activators and are mediated through signaling events downstream of NKA. Curiously, genomic deletion of PML in human cancer cells failed to abrogate the cytotoxic effects of CGs and other apoptotic stimuli such as ceramide and arsenic trioxide that were previously shown to function through PML in mice. These findings suggest that alternative pathways can compensate for PML loss to mediate apoptosis in response to CGs and other apoptotic stimuli.

## Introduction

PML was first discovered through its involvement in t(15;17) chromosomal translocations with RAR-alpha in acute promyelomonocytic leukemia (APML) [1]. PML is a predominantly nuclear protein, which seeds the formation of heterogeneous multiprotein subnuclear structures (nuclear bodies-NBs; PML oncogenic domains-POD; ND10 bodies) ranging in size from 0.2 to 1 μm with diverse functions related to the control of gene expression. At least 50 different proteins have been shown to localize to PML NBs, either constitutively or transiently, to mediate a range of processes including response to DNA-damage, cell cycle control, anti-viral response, and apoptosis[2]. Although the molecular mechanisms governing PML function and regulation are not completely understood, it is well established that PML undergoes a number of functionally important post-translational modifications in response to certain forms of cellular stress and signaling, including SUMOylation, phosphorylation, ubiquitination and acetylation [3]. SUMOylation is a form of reversible posttranslational modification that involves addition of ubiquitin-related modifier proteins called SUMO to target proteins. It is well recognized that, similar to other posttranslational modifications, SUMOylation plays crucial roles in chromatin organization, transcription, signal transduction and various other cellular processes [4]. PML SUMOylation is essential for PML NB formation and apoptosis in tumor-derived cells [2]. Arsenic trioxide induces PML SUMOylation, NB formation, and apoptosis in leukemia cells and is currently used to treat APML patients [5, 6]. In addition, PML functions as a viral restriction factor and some oncogenic viruses such as Kaposi’s Sarcoma-Associated Herpesvirus and cytomegalovirus produce proteins that disrupt NBs, in the latter case by de-SUMOylation of PML [7, 8]. Here, we show the development of a high-throughput phenotypic screen designed to identify modulators of promyelocytic leukemia protein (PML) nuclear body (NB) formation. We identified gitoxigenin as a strong inducer of PML NBs. Gitoxigenin belongs to a large family of steroid-based natural products—cardiac glycosides (CGs)–that were originally isolated from a variety of plants and animals, and have established clinical applications in the management of congestive heart failure and atrial arrhythmia [9–11]. Recent studies found that this class of structurally related compounds have potent anticancer [12] and antiviral [13–15] activities, although their clinical utility in these indications is hampered by dose-limiting cardiotoxicity. The biological activities of CGs are primarily mediated via their inhibitory binding to the catalytic α1subunit of a ubiquitous ATP-dependent ion pump, Na^+^/K^+^-ATPase (NKA), which not only blocks the sodium and potassium ion exchange across the cytoplasmic membrane, but also triggers a signaling cascade involving Src, epidermal growth factor receptor (EGFR) and phospholipase C (PLC) [16].

## Results and Discussion

**Fig 1:**
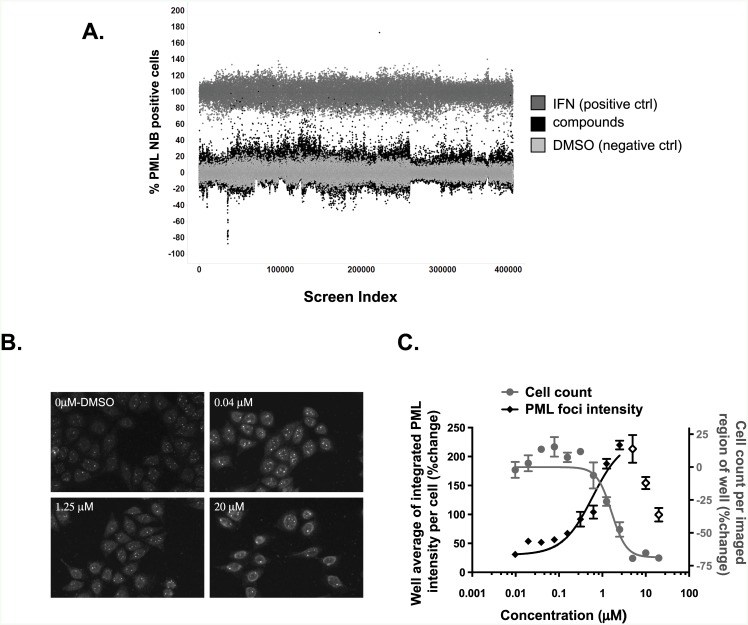
High content screening identifies cardiac glycoside as an inducer of PML nuclear body formation. **A,** Scatterplot of the data obtained from a total of 1,008 384-well library plates that were screened, imaged, and analyzed, yielding an average Z’ of 0.50 and a hit rate of 0.3%. IFN 10 U/μl (positive control, dark gray), DMSO (negative control, light gray), compounds (black) **B and C,** HeLa cells were treated with increasing concentrations of gitoxigenin as well as DMSO as a negative control for 18h. PML NB formation was determined by immunofluorescence with anti-PML antibody and was analyzed using an algorithm described in Materials and Methods. The images are representative PML NB staining of cells treated with DMSO and 0.04 μM, 1.25 μM and 20 μM gitoxigenin for 18h (B). The graphs show fitted curves for PML NB (black diamonds) and cell count (grey circles) over the full dose range of gitoxigenin (C). PML NB values at high concentrations are shown with open diamonds and are excluded from the fitted curve due to the cytotoxicity as evident from dramatic decrease in cell counts at these concentrations.

Apart from arsenic trioxide, which has several limitations as a therapeutic, very few small molecules have been reported to induce PML NB formation [17, 18]. To find novel non-arsenic inducers of PML activation, we screened 321,600 small molecules using a phenotypic high content assay. We used a monoclonal anti-PML antibody to detect endogenous PML in HeLa cells following 18 hours of treatment with compounds [19]. To measure the extent of NB formation in this large sample set, an automated detection algorithm was developed to quantify both the number of NBs per-nucleus and the percentage of nuclei per image, which achieved a threshold number of NBs (S1 Fig). A total of 1,008 384-well library plates were screened, imaged, and analyzed, yielding good overall screen statistics with an average Z’ of 0.50, average S/B of 7.4, and a hit rate of 0.3% (Fig 1A). This low hit-rate was anticipated given the highly specific phenotype being measured.

Confirmed hits from the primary screen were tested in a second cell line (PPC1) to determine whether the effects on PML were cell-type specific and additional profiling against a panel of secondary assays further validated hit compounds (S2 Fig and S1 Table).

We noted that gitoxigenin displayed a bimodal concentration response curve for PML NB formation, wherein a maximum threshold level was reached, followed by a reduction in the number of NBs detected at higher concentrations (Fig 1C). Gitoxigenin EC_50_ for NB formation is 0.6 μM, similar to its reported K_d_ of 0.5 μM for inhibition of human NKA α1 [20]. The reduction in NBs at high concentrations correlated with lower cell count, suggesting the compound might have effects on cell viability or adhesion. The cytotoxicity of gitoxigenin was verified independently using a homogeneous plate-reader assay (EC_50_ = 2.1 μM) (S2 Fig), in agreement with the general anti-proliferative activities of CGs.

**Fig 2:**
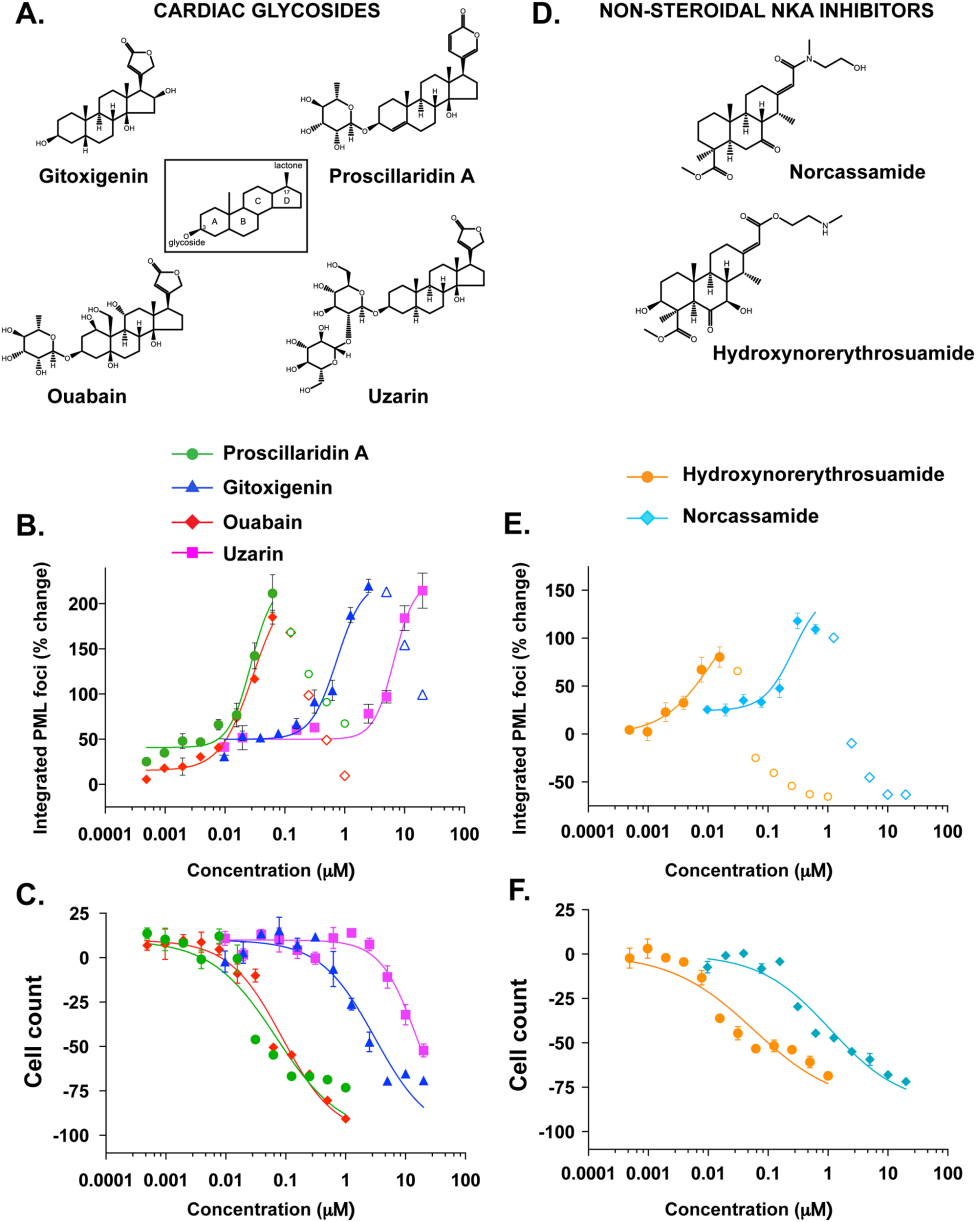
Diverse cardiac glycosides (CGs) and non-steroidal NKA inhibitors induce PML-NB formation. **A,** Structures of diverse cardiac glycosides (CGs). All CGs share a common structural motif comprised of a steroidal core adorned with an unsaturated lactone at ring position 17. CG lactone head-groups come in two varieties, unsaturated butyrolactones, such as gitoxigenin, ouabain and uzarin, and α-pyrones such as proscillaridin A. With the exception of the aglycones (e.g. gitoxigenin), the core is double substituted to contain a sugar moiety at ring position 3. **B and C,** HeLa cells were treated with increasing concentrations of CGs for 18h. PML NB formation and cell count was determined as described in Materials and Methods. **D,** Structures of non-steroidal NKA inhibitors are shown. **E and F,** HeLa cells were treated with increasing concentrations of non-steroidal NKA inhibitors for 18h. PML NB formation and cell count was determined as described in Materials and Methods. The data are means of four replicates and the error bars are SEMs. PML NB values at high concentrations are shown with open symbols in B and E and are excluded from the fitted curve due to the cytotoxicity as evident from dramatic decrease in cell counts at these concentrations (C and F).

To investigate whether gitoxigenin’s ability to induce PML NBs could be extended to other members of the same family of compounds, we evaluated a selected group of CGs with similar steroidal cores but distinct unsaturated lactone rings or sugar moieties. Their reported human NKA inhibition potencies, measured by the binding affinity (K_d_) against either the whole enzyme complex [21] or the α1 isoform subunit [20], ranged from ~0.003 μM to ~6.0 μM. We observed a striking correlation between the compound’s NKA inhibition activity and the EC_50_ of PML NB formation (Fig 2B). Potent NKA inhibitors, such as proscillaridin A and ouabain, induced NB formation in the nanomolar range whereas uzarin, a weak NKA inhibitor, required more than 100-times higher concentrations to elicit a similar effect. Additionally, the compounds exerted cytotoxicity in cells with similar potencies (Fig 2C and S2 Fig). These effects were also observed in other CGs tested (S2 Table). Such a convergent structure-activity relationship strongly suggests the entire CG family of compounds can induce the formation of PML NB to a degree proportional to their potency of NKA inhibition. We extended these observations further by testing several non-steroidal inhibitors of NKA, including two cassaine analogs, norcassamide and 3-hydroxynorerythrosuamide (Fig 2D) [22]. Consistent with the hypothesis that CG-induced PML NB formation is mediated through NKA, these non-steroidal compounds also induced PML NB formation and cell death with similar relative potencies (Fig 2E and 2F, and S2 Fig and S2 Table). Interestingly, the effects of CGs and cassaines on PML NB formation were not mimicked by siRNA-mediated knock-down of NKA. Although siRNA of α1 resulted in significant reduction in protein as measured by immunocytochemistry and immunoblot, no statistical increase in PML NBs was detected (S4 Fig).

**Fig 3:**
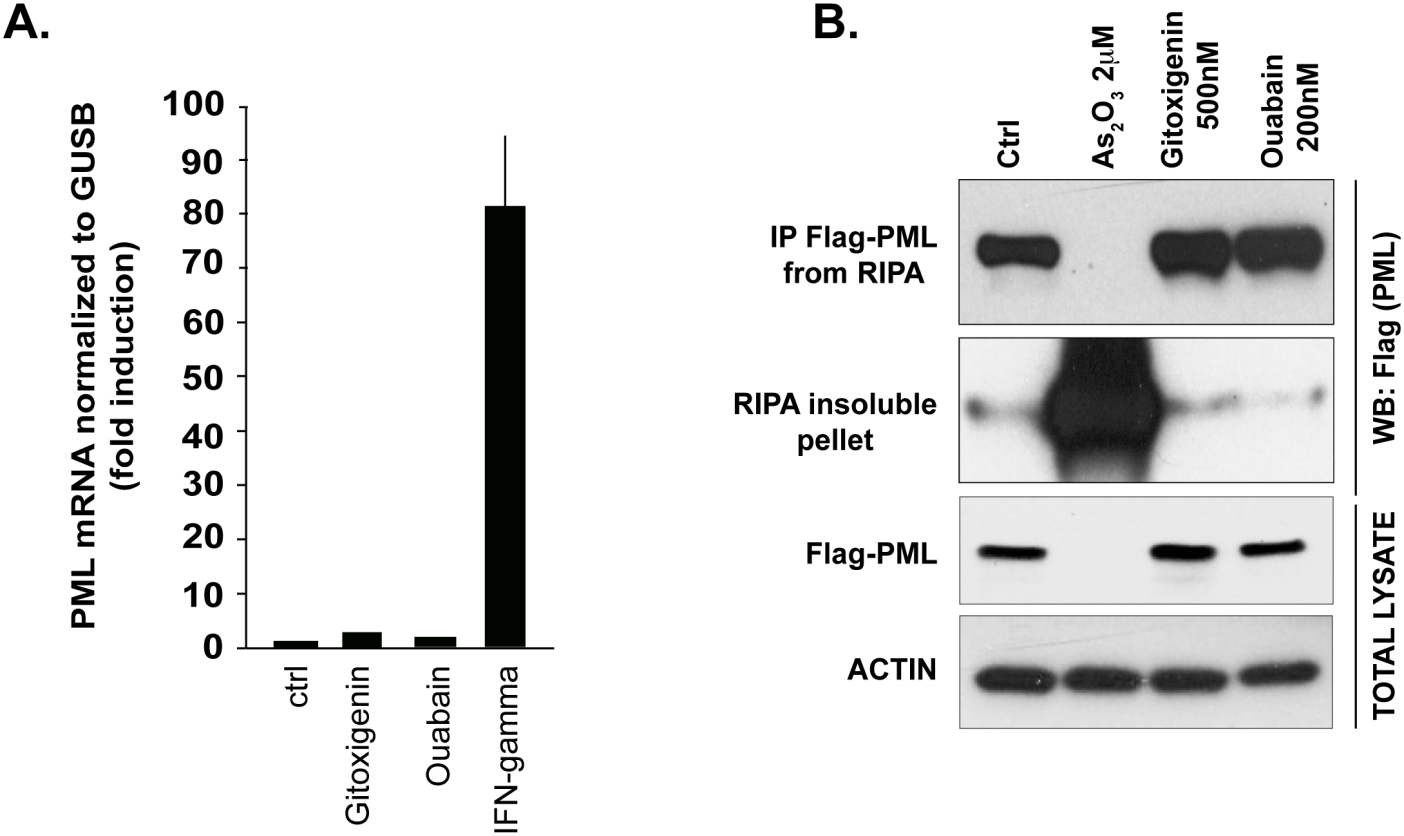
CGs do not induce PML expression or association with nuclear matrix. **A,** HeLa cells were dispensed into 384-well pates at a density of 2000 cells/wells. The next day the cells were incubated with 2 μM gitoxigenin, 200 nM ouabain and 16 u IFNϒ for 18h. RNA was isolated and RT-qPCR was performed for PML and GUSB. PML expression was normalized to GUSB internal control. Error bars are SEMs of 6 replicates. **B,** HEK293T were transfected with Flag-PML IV. The next day the cells were treated as indicated for 24h, followed by lysis in RIPA buffer and centrifugation. RIPA supernatants were immunoprecipitated using anti-Flag antibody. The total lysates before immunoprecipitation, the immunoprecipitated complexes and the RIPA-insoluble pellets were separated on SDS-PAGE followed by immunoblotting with anti-Flag (PML) or anti-ACTIN antibody.

**Fig 4:**
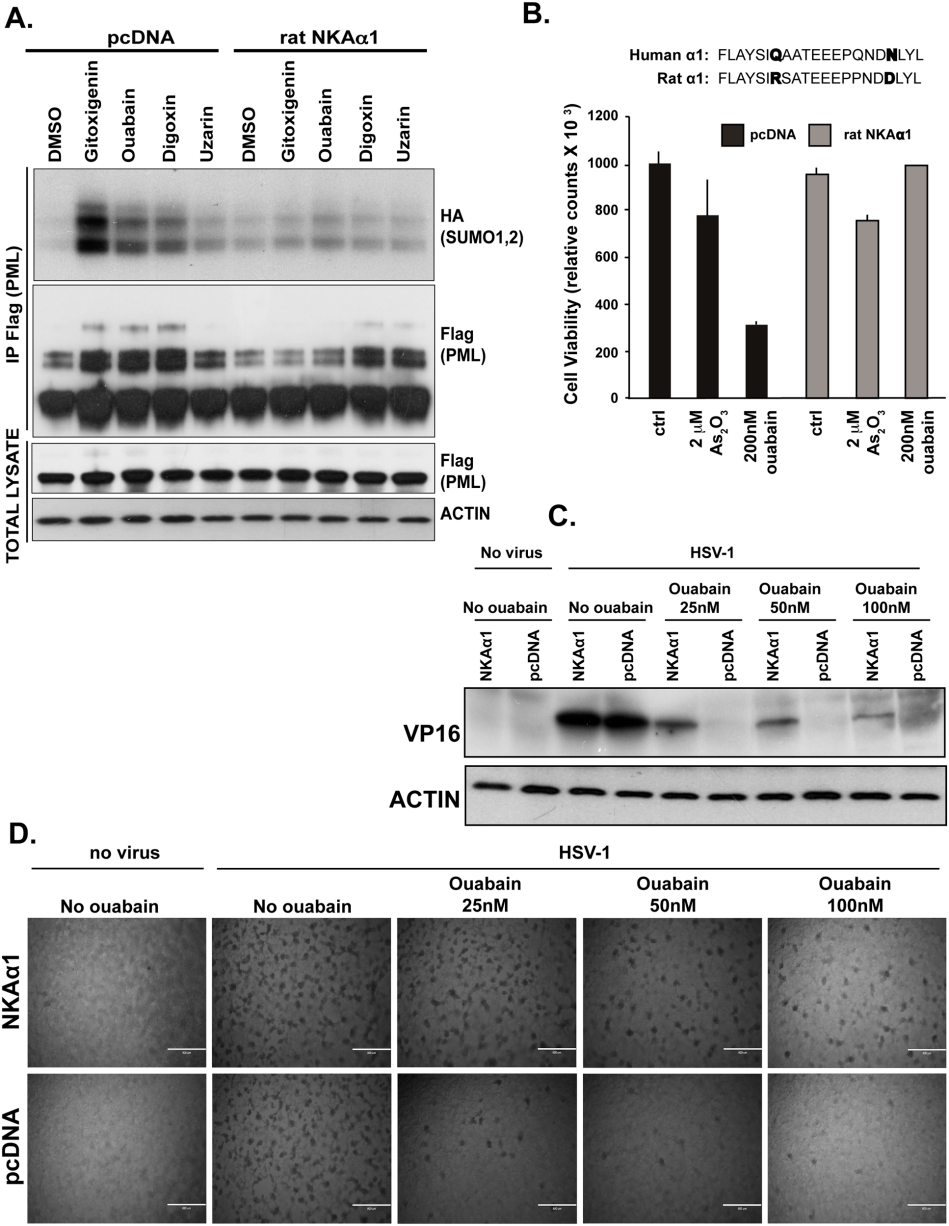
CGs effects on PML SUMOylation, cell survival and anti-viral effects are mediated by NKA α1. **A,** HEK293T cells were transfected with plasmids encoding HA-Sumo-1, HA-Sumo-2 and Flag-PM IV together with either empty vector or rat NKAα1. The next day the cells were treated as indicated for 24h, followed by lysis and IP using anti-Flag (PML) antibody. The total lysates before immunoprecipitation and the IP-ed complexes were separated on SDS-PAGE followed by immunoblotting with anti-Flag (PML), anti HA (Sumo-1 and Sumo-2) or anti-ACTIN antibodies. **B,** Human NKA subunit α1 contains residues Q118 and N129, which are essential for CG binding. Rat NKAα1 lacks these critical residues, rendering it insensitive to CG. HEK293T cells were transfected with either empty vector or rat NKA α1. The following day the cells were treated as indicated for 48h, followed by cell viability determination using Cell Titer Glo assay. Data are means of three replicates and the error bars are standard deviations. **C,** HEK293T cells were transfected with either NKAα1 or pcDNA. The following day, the cells were recovered and dispensed into 24-well plates and the day after they were pre-treated with 0, 25 nM, 50 nM or 100 nM ouabain for 5h, followed by infection with HSV-1 KOS for 24 hrs. The produced virus was harvested for infection of naïve cells in D, and was detected by immunoblotting with anti-VP16 antibody. **D,** Phase-contrast image of HEK293T cells 48h after infection with HSV-1 virus produced in cells as described in C. Less virus was produced in cells treated with ouabain, as evident from less VP16 expression (C) and less cell killing (D). The scale in D represents 800 μm. The effect of ouabain was reversed with overexpression of rat NKAα1.

The induction of PML NB formation could be a consequence of increased PML transcription and/or post-transcriptional effects. For example, interferons induce transcription of PML, resulting in elevated PML protein levels and subsequent NB formation [2, 23], while arsenic does not affect PML transcription but induces SUMOylation of PML, which is required for proper assembly of NB complexes [24]. We did not detect any increase in PML transcripts by quantitative RT-PCR at time-points that induce NB formation in HeLa cells (Fig 3A). However, all CGs tested were found to induce PML SUMOylation at concentrations approximating their EC_50_ values for PML NB formation and cytotoxicity, with the two more potent compounds, ouabain and digoxin, working at 0.05 μM and 0.1 μM respectively, and uzarin showing a very modest effect at 5.0 μM (Fig 4A). Although CGs share this effect of PML SUMOylation with arsenic, we noted that unlike arsenic, CGs do not redistribute PML into the insoluble nuclear matrix fraction, which suggests a distinct mechanism of action (Fig 3B).

Although mammalian NKA α1 subunits are very sensitive to the inhibitory binding of CGs, two amino acid differences between the rodent and human NKA α1 subunit’s first extracellular loop renders the rodent-derived transporter virtually insensitive to binding and modulation by CGs, thereby rendering rodent cells resistant to CGs [12]. Consistent with this finding, we observed that CGs, which exhibit various cytotoxicities in human cells, induced neither increased PML NB formation nor cytotoxicity in murine IMDC-3 cells (S3 Fig).

To further verify that the induction of PML NB formation by CGs was dependent upon the inhibition of the human NKA, we overexpressed the rat CG-insensitive NKA α1 in human HEK293T cells. Strikingly, overexpression of rat NKA α1 abrogated CG-induced PML SUMOylation as compared to cells transfected with vector alone (Fig 4A). Moreover, overexpression of this CG-insensitive form of NKA α1 suppressed the cytotoxic effects of ouabain, while it had no effect on arsenic trioxide-induced toxicity in HEK293T cells as it functions independently of NKA (Fig 4B). Consistent with PML’s role in inducing cell death, we found that overexpression of Flag-PML IV reduces viability of HEK293T cells in a dose-dependent manner (S6 Fig), as was previously reported for other cells [25, 26].

Given the well-established role of PML SUMOylation and NB formation in anti-viral defense [24, 27], we tested the ability of ouabain to inhibit HSV-1 infection of human neural cells (HEK293T), in which we found ouabain-induced PML SUMOylation (Fig 4A). Consistent with previous reports, we observed that ouabain rescued HEK293T cells from HSV-1 infection [13]. At concentrations ranging from 0.025 to 0.100 μM, ouabain reduced the ability of HEK293T cells to replicate HSV-1 virus and blocked expression of the viral gene VP16 (Fig 4C). In addition, ouabain-treated cells showed reduced ability to produce infectious HSV-1 virulent particles as evidenced by their reduced ability to infect and kill naive HEK293T cells (Fig 4D). Similar anti-viral effects of ouabain were observed in Vero cells (S7 Fig). Importantly, as was noted for effects of GCs on PML and cell viability, the anti-viral actions of ouabain were abrogated by overexpression of the CG-insensitive rat NKA α1 subunit (Fig 4C and 4D). These findings are consistent with the hypothesis that the recently ascribed anti-viral activity of CGs is mediated by signaling through NKA to the PML viral restriction factor.

**Fig 5:**
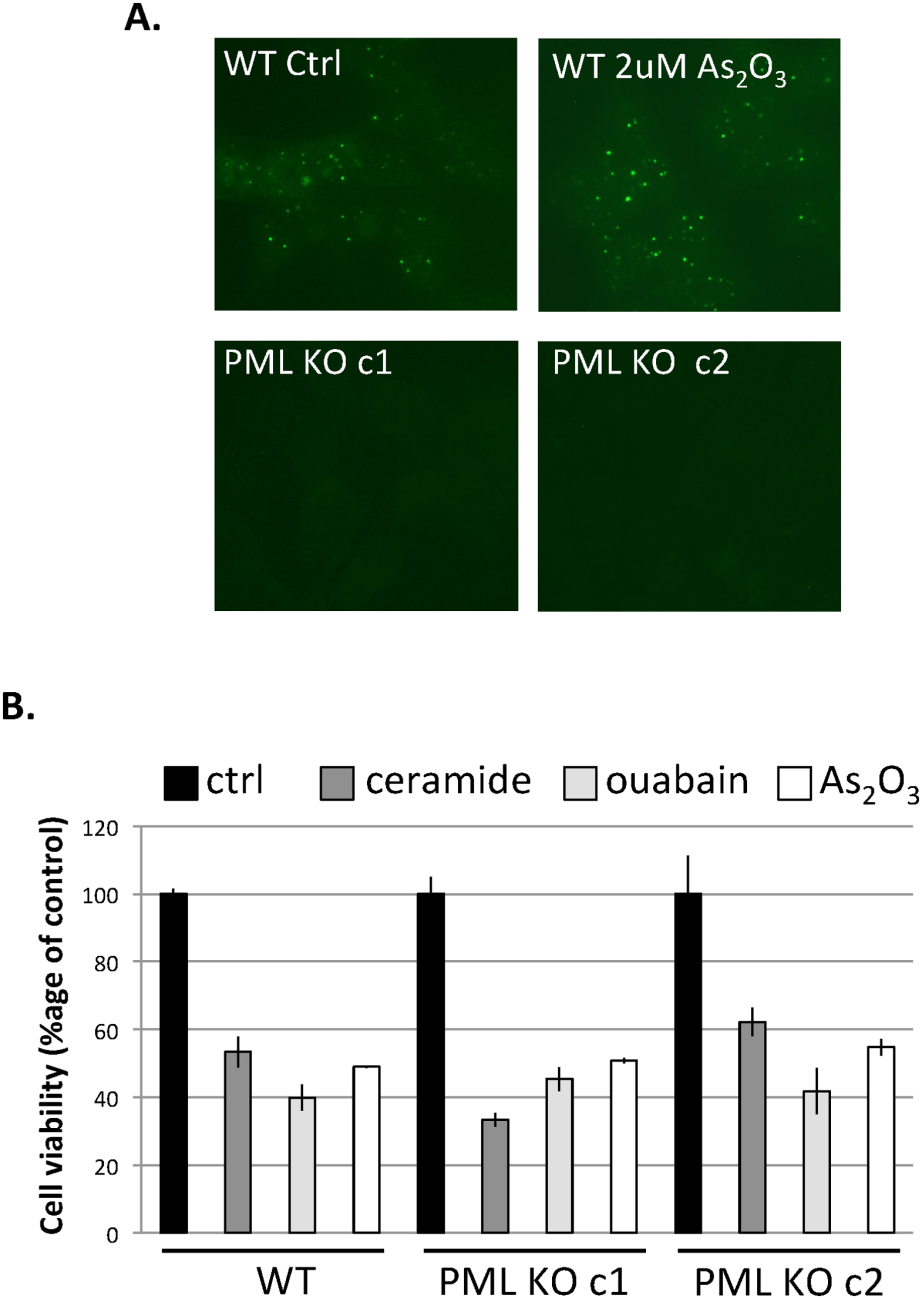
Loss of PML does not attenuate cell death induced by apoptotic stimuli. Two *PML* knockout HEK293T cell clones (PML KO c1 and c2) were generated by CRISPR/Cas9 approach. **A,** PML protein levels were determined by immunofluorescence using anti-PML antibody in the control and 2 μM arsenic trioxide (As_2_O_3_)-treated wild type HEK293T (WT), as well as in two PML knockout clones (PML KO c1 and c2). **B,** Wild-type and knockout clones were cultured in 96 well plates at a density of 15,000/well. The next day, the cells were treated with 60 μM C2-ceramide, 300nM ouabain or 5 μM arsenic trioxide for 48h. Cell viability was determined using Cell Titer Glo assay. Data are mean ± SEM (n = 3).

To determine if PML is necessary for mediating these CG-induced effects, we generated *PML* gene knockout cells using the CRISPR/Cas9 gene editing approach. Since *PML* generates eight splice variants, we targeted exon 3 and exon 4, which are common to all variants. We used three different guide RNAs to direct Cas9 to produce multiple nicks in the *PML* gene. We generated two different *PML* gene knockout clones that have parts of *PML* genomic sequence excised and deleted by Cas9. *PML* KO clone 1 has 170 bps of exon 3 deleted in one allele as well as most of exon3, intron 3 and exon 4 deleted in the other allele. *PML* KO clone 2 has deletion of most of exon3, intron 3 and exon 4 in both alleles. Immunofluorescence labeling with anti-PML antibody showed typical punctate staining of PML protein in the wild type HEK293T cells, which was enhanced following 1h treatment with 2 μM arsenic trioxide, as previously reported [28]. However, both *PML* KO clones were completely deficient for PML protein staining either in the untreated cells (Fig 5A) or in the arsenic-treated cells (data not shown). Previous studies using cells derived from *pml* gene knockout mice showed that PML is partially responsible for apoptosis induced by various cytotoxic agents such as ceramide and arsenic trioxide[29, 30]. However, we did not detect any difference in cell death induced by ceramide, arsenic trioxide or cardiac glycosides in the CRISPR/Cas9-generated *PML* knockout human cell lines (Fig 5B). In addition, our data shows that arsenic trioxide reduces cell viability by 50% in 48h, while it has previously been shown that arsenic trioxide induces NB formation within hours followed by degradation of most PML isoforms in 24h [31]. The precise mechanism of how arsenic trioxide induces cell death is not clearly understood and various mechanisms have been proposed in different cell types including apoptosis, reactive oxygen species, autophagy and others [32]. Our results suggest the existence of redundant pathways and/or macromolecular structures that can compensate for the loss of PML in HEK293T cells. Similar alternative pathways may exist in cells derived from *pml* knockout mice and may be highly cell-type specific. It was previously shown that the loss of PML had only a partial effect on arsenic-induced cell death of PML null mouse splenocytes and thymocytes [30], while PML loss had no effect on arsenic-induced cell death in *pml* null MEFs [33]. Similar to arsenic trioxide, CGs may induce cell death through alternative pathways in the absence of PML. Further studies would be required to elucidate the contribution of alternative pathways in CG-mediated apoptosis in various cell types.

The chemical genetic and reverse genetic approaches described herein provide a molecular link between NKA and PML, two previously unrelated cellular pathways. However, the precise signaling mechanism downstream of NKA remains to be elucidated. While inhibition of the ATP-dependent inotropic activity by NKA inhibitors could account for the effects observed on PML, we did not detect a statistical increase in PML NB formation by siRNA-mediated knockdown of NKA in human cells (S4 Fig). These data suggest that CG-mediated effects on PML are not simply a consequence of reduced Na^+^/K^+^ flux. This is consistent with a previous report showing ouabain-induced cytotoxicity was Na^+^/K^+^-independent [34] although another group reported contrasting data for digoxin [14]. We favor a model wherein NKA α1 subunit binding of CGs elicits downstream signaling, potentially via the Src/EGFR or PLC complex or via yet unidentified partner, resulting in PML SUMOylation and NB formation. Overexpression of the rodent CG-insensitive transporter may titrate the signaling components away from the CG-binding competent human transporter. Other components of the NKA-PML signaling pathway may be absent in rodents since we were unable to induce CG-mediated cytotoxicity in rodent cells transfected with the human NKA α1 subunit (data not shown). The discovery that CGs induce PML activation via inhibition of NKA lays the foundation for future mechanistic studies and provides the potential to identify modulators of NKA, which activate PML without associated cardiotoxicity.

## Materials and Methods

### Cell Culture

HEK293T, HeLa, Vero and mIMDC-3 cells were grown in DMEM medium with 10% fetal bovine serum (FBS), 2 mM glutamine, 100 U/mL penicillin and 100 μg/mL streptomycin. PPC-1 cells were grown similarly in RPMI medium. All cell lines were obtained from ATCC.

### Library screening

Sanford-Burnham’s internal HTS library containing 321,600 chemically diverse compounds with good physicochemical properties was selected for screening HeLa cells for PML nuclear body (PML NB) formation as described below. DMSO (0.1%) was used as a negative control and 10 U/μl interferon γ was used as a positive control for PML NB formation. Compounds and DMSO were transferred by acoustic dispense using Echo555 (LabCyte).

### PML Immunofluorescence assay

For the primary HCS campaign, compounds in 100% DMSO were pre-dispensed into 384-well assay plates using an acoustic dispenser followed by addition of 10 μl of media. 4,000 cells/well in 30 μl volume per well were added to the assay plates, for a final assay compound concentration of 10 μM in 0.1% DMSO, and incubated for approximately 18 hours at 37C (5% CO_2_). After compound incubation, the cells were washed with phosphate-buffered saline (PBS), fixed with 4% formaldehyde for 25 min, washed, permeabilized with 0.1% Triton X-100 for 5 min, washed, incubated with the mouse monoclonal anti-human PML primary antibody (PG-M3, Santa Cruz Biotechnology) diluted 1:400 in 5% bovine serum albumin (BSA) for 1 h, washed, incubated with the Alexa Fluor 488 chicken anti-mouse IgG secondary antibody (Invitrogen) diluted 1:400 in 5% BSA for 1 h, washed, and placed in a 100-ng/ml DAPI-PBS solution (Invitrogen) overnight. Each wash step was a 3x fluid exchange using a Titertek Map-C microplate washer.

For hit confirmation and secondary assays, the above described immunofluorescence protocol was applied except that cells were dispensed first into assay plates and allowed to attach overnight, followed by compound addition the next day. For phospho-H2AX and phosphor-Chk1 immunostaining, a rabbit polyclonal anti-human phospho-histone H2A.X (Ser139; diluted 1:400; Cell Signaling Technology) and a rabbit polyclonal anti-human phospho-Chk1 (Ser317; diluted 1:400; Cell Signaling Technology) primary antibody were used with an Alexa Fluor 568 goat anti-rabbit IgG secondary antibody (Invitrogen).

### High content imaging

Plates were imaged using an Opera QEHS high content screening system with a 20X 0.45NA ELWD Plan Fluor (air) objective (3 image fields/well, ~200 cells/well). The images were analyzed using the PML detection protocol (S1 Fig), which was developed based on a custom Acapella (PerkinElmer) nuclei and spot detection algorithm. A total of 46 analysis parameters were extracted from the images, including 12 parameters related to DAPI-channel nuclei morphology and fluorometry, 12 parameters related to AlexaFluor488 channel cell morphology and fluorometry, 19 parameters related to spot detection under nuclei mask, 1 parameter overall nuclei (cell) count, and 2 parameters derived from nuclei parameters (small & bright nuclei): number of “dying cells”, and percentage “dying cells”. Images and data were uploaded to the Columbus image-data management system (PerkinElmer). For the primary HCS campaign, the “Percentage of PML-Foci Positive Cells” (% of cells with higher than baseline number of foci) was used as primary assay read-out. For confirmation and secondary assays, multiple foci related assay read-outs were evaluated and “well average of number of foci per cell” or “well average of integrated foci staining intensity per cell” were utilized depending on the assay.

### Statistical Analysis

Single concentration primary screen, dose response confirmation, and secondary assay data were further analyzed using Genedata Screener Analyzer software. Plate data was quality-controlled for acceptable cell counts and assay performance. Plates with Z’-values <0.4 for the primary assay read-out were flagged for repeat and removed from further analysis. Wells with <50 cells per well were flagged as low cell count / cytotoxic. Raw numbers of cell counts per well and foci counts per cell were tracked for quality control.

Overall primary HTS assay performance was evaluated resulting in an average Z’ for all plate batches of 0.50, a robust Z’ of 0.54 (RZ’ uses median and median absolute deviation for calculation) and a signal to background ratio (S/B) of 7.44.

### Immunoprecipitation and western blotting

For analysis of PML SUMOylation, HEK293T cells were plated at a density of 1.5 million per 10cm plate and the next day they were transfected with 5μg of Flag-PML-IV, 2.5μg of HA-SUMO-1, 2.5μg HA-SUMO2 and 5μg either pcDNA vector or rat NKAα1. 24 h after the transfection, the cells were treated with 1.5μM gitoxigenin, 50nM ouabain, 100nM digoxin and 5μM uzarin for 6 h. The cells were lysed in NP40 buffer and PML was immunoprecipitated with anti-Flag antibody, washed 3 times and resolved on SDS-PAGE. Immunoblotting was done using anti-HA antibody and anti-Flag antibody. For detection of NKA α1, VP-16 and actin, the following antibodies were used: NBP1-61321 (Novus Biologicals), ab110226 (abcam) and sc-1616 (Santa Cruz Biotechnology), respectively.

### Quantitative PCR

HeLa cells were plated at a density of 2000 cells/384 wells. The next day the cells were incubated with 2μM gitoxigenin, 200nM ouabain and 16U/ml IFNϒ for 18 h. RNA was isolated using Qiagen RNA mini kit and the RT-qPCR was performed using RNA-to-Ct kit (Invitrogen). Taqman primers were used to determine the expression of PML and a housekeeping gene GUSB. The expression of PML was normalized to GUSB.

### HSV-1 viral infection

HSV-1 KOS was a kind gift from Dr. Carl Ware from Sanford Burnham Prebys. HEK293T cells were plated at 50% confluency and the next day they were transfected with 15μg of either NKAα1 or pcDNA. The following day, the transfected cells were re-plated into 24 wells at 30% confluency. One day after re-plating, the cells were pre-treated with 0, 25, 50 or 100nM ouabain for 5h, followed by infection with 1 MOI of HSV-1 KOS for 24 h. The produced virus was harvested by 2 freeze-thaw cycles of media and cells. Part of this lysate was mixed with 2X sample loading buffer and was run on SDS-PAGE followed by immunoblotting with anti-VP16 and anti-actin antibody. In addition, one tenth of the produced virus was added to naïve HEK293T cells plated at 70% confluency in 24-well plates. The cells were incubated for 48 h before the pictures were taken.

### Generation of PML KO clones

Three different Crispr/Cas9 knockout plasmids containing guide RNA sequences directed against exon 3 (GCGGTACCAGCGCGACTACG and CTGCGCGTGAACCGCGCCAA) and exon 4 (CTCACCAGGTCAACGTCAAT) of the human *PML* gene were purchased from Santa Cruz Biotechnology (sc-400145). The mix of three plasmids were transfected into HEK293T cells using Lipofectamine 2000. Three days following transfection, single cells were sorted by FACS into 96 well plate based on the high expression of GFP that is encoded by CRISPR/Cas9 knockout plasmids. The clones were grown in 96 wells for two weeks, and were then further expanded in 6 well plates. Genomic DNA was extracted from each clonal population and the Cas9-targeted region of *PML* was amplified by PCR. The size of the amplified PCR product was used to identify the clones that were nicked by Cas9 at two different sites resulting in the loss of genomic regions between the two nicked sites. Two such clones were identified. *PML* KO clone 1 had an had a deletion between two sites in exon 3 in one allele, and a deletion between exon 3 and exon 4 in the other allele. *PML* KO clone 2 had a deletion between exon 3 and exon 4 in both alleles (S5 Fig). The lack of PML protein expression was determined by immunofluorescence using two different anti-PML antibodies from Santa Cruz Biotechnology, sc-966 (Fig 5A) and sc-5621 (data not shown).

## Supporting Information

S1 Appendix Supplementary Materials and Methods. (DOCX)

S1 Fig PML NB detection algorithm. To quantify the extent of PML NB formation in a high-throughput manner, an automated analysis algorithm was developed to extract 19 parameters related to PML NB formation from the images in addition to several other parameters related to cell health and general DAPI or AlexaFluor488 staining intensity. Cell detection and quantification was performed as follows. **A,** Overlay of DAPI and Alexa 488 channels **B,** Gray scale raw image obtained from Alexa 488 channel **C,** Gray scale raw image obtained from DAPI channel **D,** Nuclei detection using the DAPI channel **E,** Cell detection using detected nuclei and Alexa 488 raw image **F,** Spot detection under the nuclei region using Alexa 488 raw image **G,** Cell quantification and metrics for both DAPI and Alexa 488 channel **H,** Remove dying cells from cell population and calculate percentages of foci positive cells **I,** Calculate cell population statistics for each well **J,** The number of NBs per-nucleus and the percentage of nuclei per image that achieved a threshold number of NBs, are shown in the example analysis of interferon γ treatment (circles and triangles, respectively).
(TIFF)

S2 Fig NKA inhibitors induce cell death in HeLa cells. HeLa cells were dispensed into 384 well plates at 3000 cells/well and the next day, they were treated with increasing concentrations of cardiac glycosides (**A**) or non-steroidal NKA inhibitors (**B**) for 18h followed by cell viability assay using Cell Titer Glo. Data are means of three replicates and the error bars are standard deviations.
(TIFF)

S3 Fig Rodent cells are insensitive to NKA inhibitors. Murine IMDC-3 cells were treated with increasing concentrations of NKA inhibitors for 18h. PML NB formation (**A**), cell counts (**B**) and cytotoxicity (**C**) were determined as described in Material and Methods. Data are means of three replicates.
(TIFF)

S4 Fig NKAα1 knockdown does not induce PML NB formation. **A,** PPC-1 cells were seeded into 6 well plates at 200,000 cells/well. The next day the cells were transfected with 30 nM of control siRNA or 10, 15 and 30 nM of siRNA directed against human NKAα1. At 48h post transfection, the cells were fixed and stained with anti-PML antibody or DAPI. **B,** PPC-1 cells were cultured in 6-well plates at 200,000/well. The next day they were transfected as in **A**, and at 48h post-transfection the levels of NKAα1 and actin were determined by immunoblotting using anti-NKAα1 and anti-actin antibodies.
(TIFF)

S5 Fig Generation of the *PML* KO clones. The scheme of *PML* genomic region, the sites targeted by the three guide RNAs and the genomic primers used to amplify 2504 bp region of the *PML* gene in the wild type (WT) HEK293T cells are shown. The lower panel shows the PCR amplification of the WT cells (2504bp), *PML* KO clone 1 (2334bp resulting from the excision between guide RNA 1 and guide RNA 2 and 450bp resulting from the excision between guide RNA 1 and guide RNA 3) and *PML* KO clone 2 (450bp resulting from the excision between guide RNA 1 and guide RNA 3).
(TIFF)

S6 Fig Overexpression of PML IV reduces the viability of HEK293T cells. HEK293T cells were plated at a density of 5,000 cells/well in a 96 well plate. The cells were transfected with increasing amount of either empty vector pcDNA or Flag-PML IV (18-150ng/well). 48h after the transfection, the cell viability was assessed using Cell Titer Glo. Data are means of three replicates and the error bars are standard deviations.
(PDF)

S7 Fig Ouabain rescues HSV-1-induced cell death in Vero cells. Vero cells were plated in Plate 1 at 90% confluency and were pre-treated with 0, 25, 50 or 100nM Ouabain for 5h, followed by infection with HSV-1 KOS for 24 hrs. The produced virus was harvested by 2 freeze-thaw cycles of media and cells in Plate 1. Then, one tenth of the produced virus was added to a new Plate 2 of Vero cells (70% confluent) and the cells were incubated for 48h before the pictures were taken (Note that Plate 2 was not treated with Ouabain).
(PDF)

S1 Table Testing funnel of the hits obtained from the primary screen. The hits obtained in the primary screen were confirmed at two concentrations (10 μM for hit compounds based on PML activity, 1 μM for compounds showing significant cytotoxicity at 10 μM with increased PML activity in the remaining attached cells). Confirmed hits were tested in a funnel of secondary assays (phospho-H2AX staining, phospho-Chk1 staining, cytotoxicity dose-response, PML NB dose-response) to further eliminate artifacts. Additional cell line PPC-1 was also tested in cytotoxicity and PML NB assays.
(DOCX)

S2 Table Steroidal and non-steroidal NKA inhibitors activity. EC50s for PML NB formation and cytotoxicity of various NKA inhibitors are determined following 18h treatment of HeLa and PPC-1 cells as described in Materials and Methods. The previously reported Kds for inhibition of NKAα1β1 by these compounds are also shown. NCI-60 means of GI_50_s are means of compound concentrations required to inhibit 50% of cell growth in a panel of 60 cancer cell lines as described in Materials and Methods.
(DOCX)
