# Supplementary material for: Cardiac Glycosides Activate the Tumor Suppressor and Viral Restriction Factor Promyelocytic Leukemia Protein (PML)
Source: PLoS One. 2016 Mar 31;11(3):e0152692. doi: 10.1371/journal.pone.0152692 (PMC4816303; doi:10.1371/journal.pone.0152692)
Supplement: S1 Appendix — (DOCX) [file pone.0152692.s001.docx]

**Supplementary Materials and Methods:**

***siRNA treatment***

HeLa cells were plated at 20,000 cells/96 well. The next day the cells were transfected with JetPRIME reagent (PolyPlus transfection) and either control siRNA or siRNA directed against human NKAα1 (cat no. sc-37007 and sc-36010, Santa Cruz Biotechnology). 48 h post transfection, the cells were profiled for PML NB formation by immunofluorescence staining and NKA α1 expression was determined by western blotting.

***NCI-60 means of GI_50_s***

The human tumor cell lines of the cancer screening panel were grown in RPMI 1640 medium containing 5% fetal bovine serum and 2 mM L-glutamine. For a typical screening experiment, cells were inoculated into 96 well microtiter plates in 100 µL at plating densities ranging from 5,000 to 40,000 cells/well depending on the doubling time of individual cell lines. 24h after plating various concentrations of the test compounds were added for additional 48h, followed by cell fixing in TCA (10% final concentration), washing, and staining the cellular proteins with Sulforhodamine B (0.4% w/v in 1% acetic acid) for 10min. After staining, unbound dye was removed by washing five times with 1% acetic acid and the plates were air dried. Bound stain was subsequently solubilized with 10 mM trizma base, and the absorbance was read on an automated plate reader at a wavelength of 515 nm. Additional experimental details can be found on NCI/NIH Developmental Therapeutics Program web site: <http://dtp.nci.nih.gov/branches/btb/ivclsp.html>.
