# Supplementary figures and images for: Cardiac Glycosides Activate the Tumor Suppressor and Viral Restriction Factor Promyelocytic Leukemia Protein (PML)
Source: PLoS One. 2016 Mar 31;11(3):e0152692. doi: 10.1371/journal.pone.0152692 (PMC4816303; doi:10.1371/journal.pone.0152692)

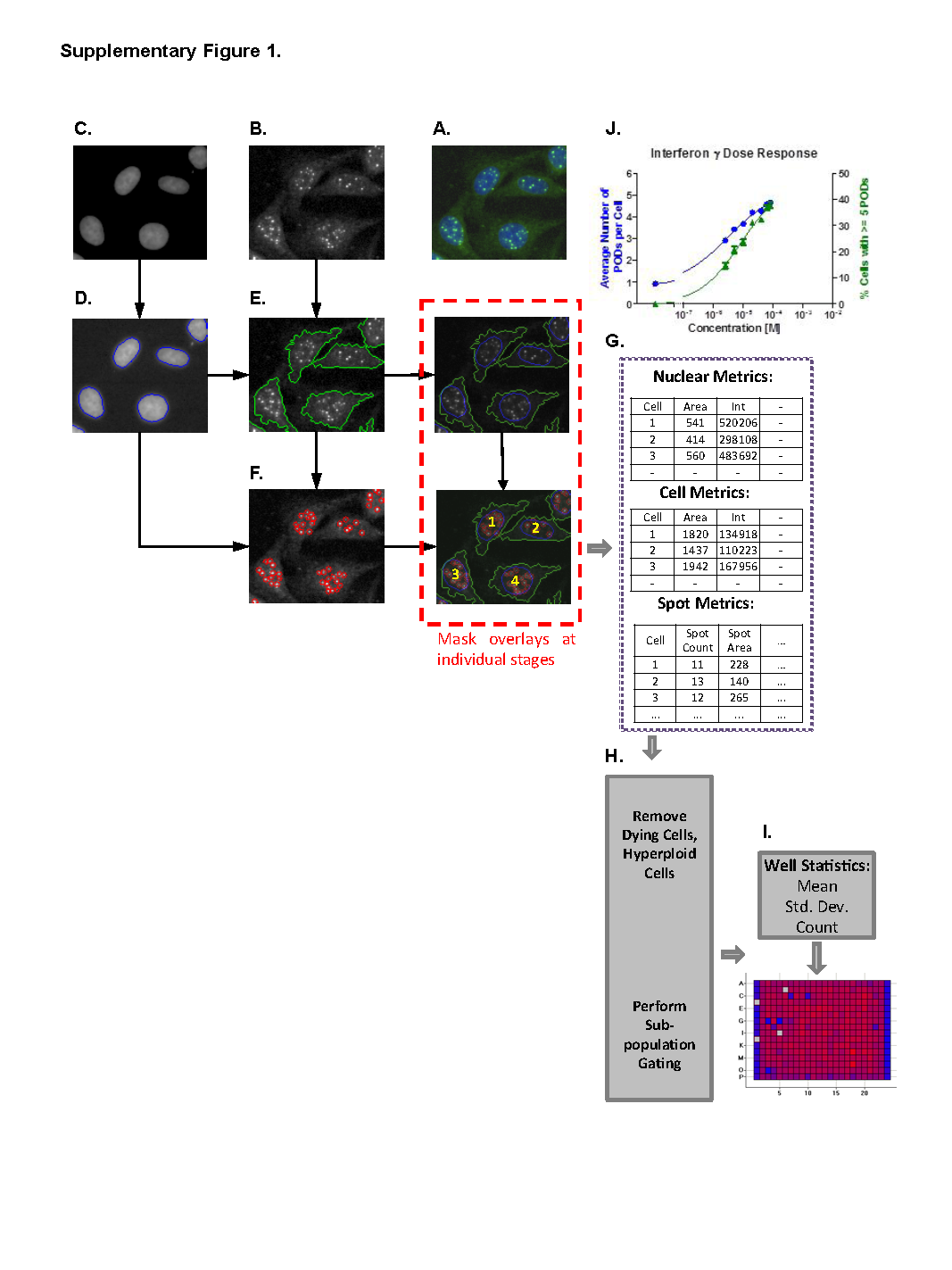

Supplement: S1 Fig — To quantify the extent of PML NB formation in a high-throughput manner, an automated analysis algorithm was developed to extract 19 parameters related to PML NB formation from the images in addition to several other parameters related to cell health and general DAPI or AlexaFluor488 staining intensity. Cell detection and quantification was performed as follows. A, Overlay of DAPI and Alexa 488 channels B, Gray scale raw image obtained from Alexa 488 channel C, Gray scale raw image obtained from DAPI channel D, Nuclei detection using the DAPI channel E, Cell detection using detected nuclei and Alexa 488 raw image F, Spot detection under the nuclei region using Alexa 488 raw image G, Cell quantification and metrics for both DAPI and Alexa 488 channel H, Remove dying cells from cell population and calculate percentages of foci positive cells I, Calculate cell population statistics for each well J, The number of NBs per-nucleus and the percentage of nuclei per image that achieved a threshold number of NBs, are shown in the example analysis of interferon γ treatment (circles and triangles, respectively). (TIFF) [file pone.0152692.s002.tiff]

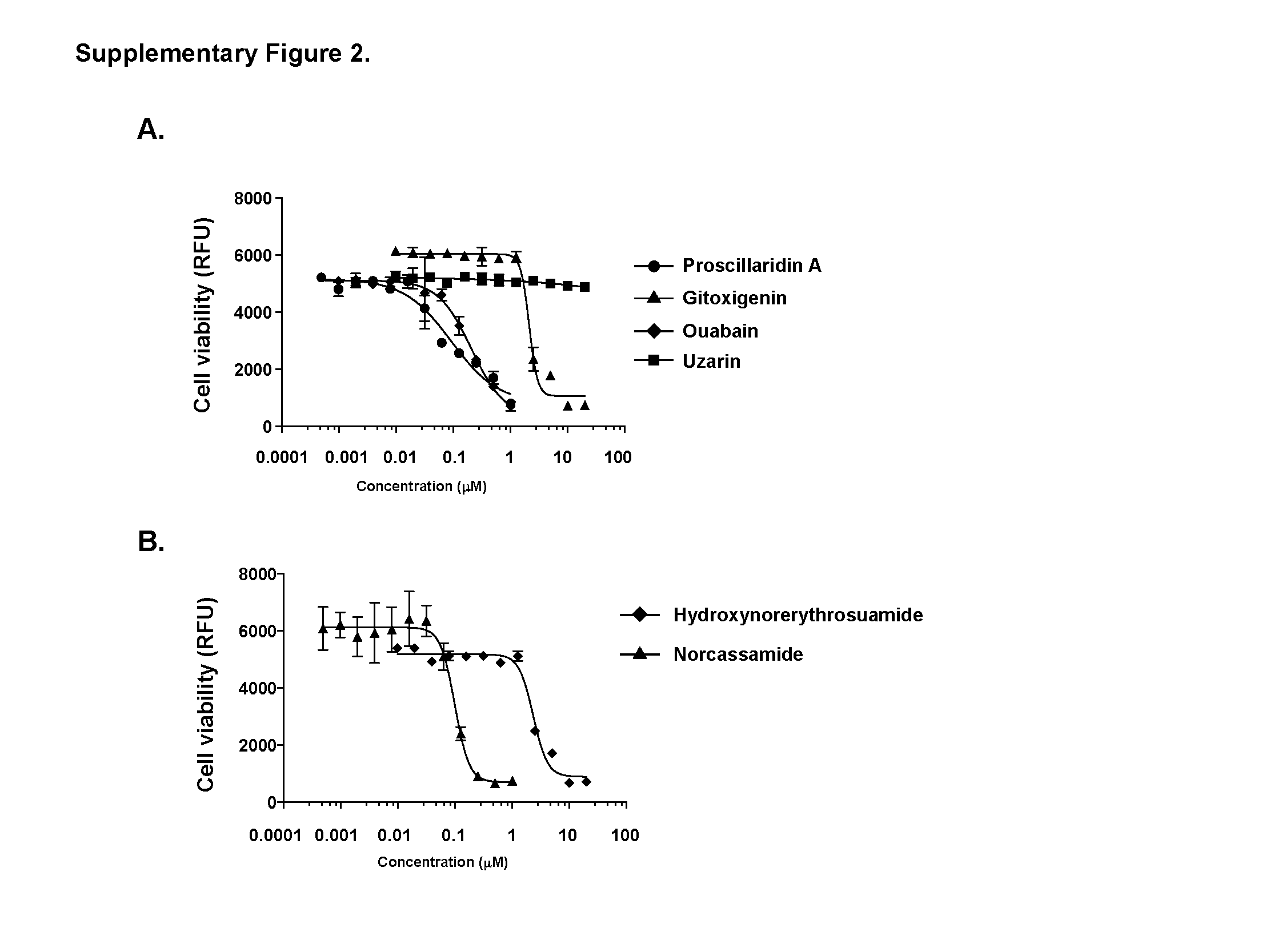

Supplement: S2 Fig — HeLa cells were dispensed into 384 well plates at 3000 cells/well and the next day, they were treated with increasing concentrations of cardiac glycosides (A) or non-steroidal NKA inhibitors (B) for 18h followed by cell viability assay using Cell Titer Glo. Data are means of three replicates and the error bars are standard deviations. (TIFF) [file pone.0152692.s003.tiff]

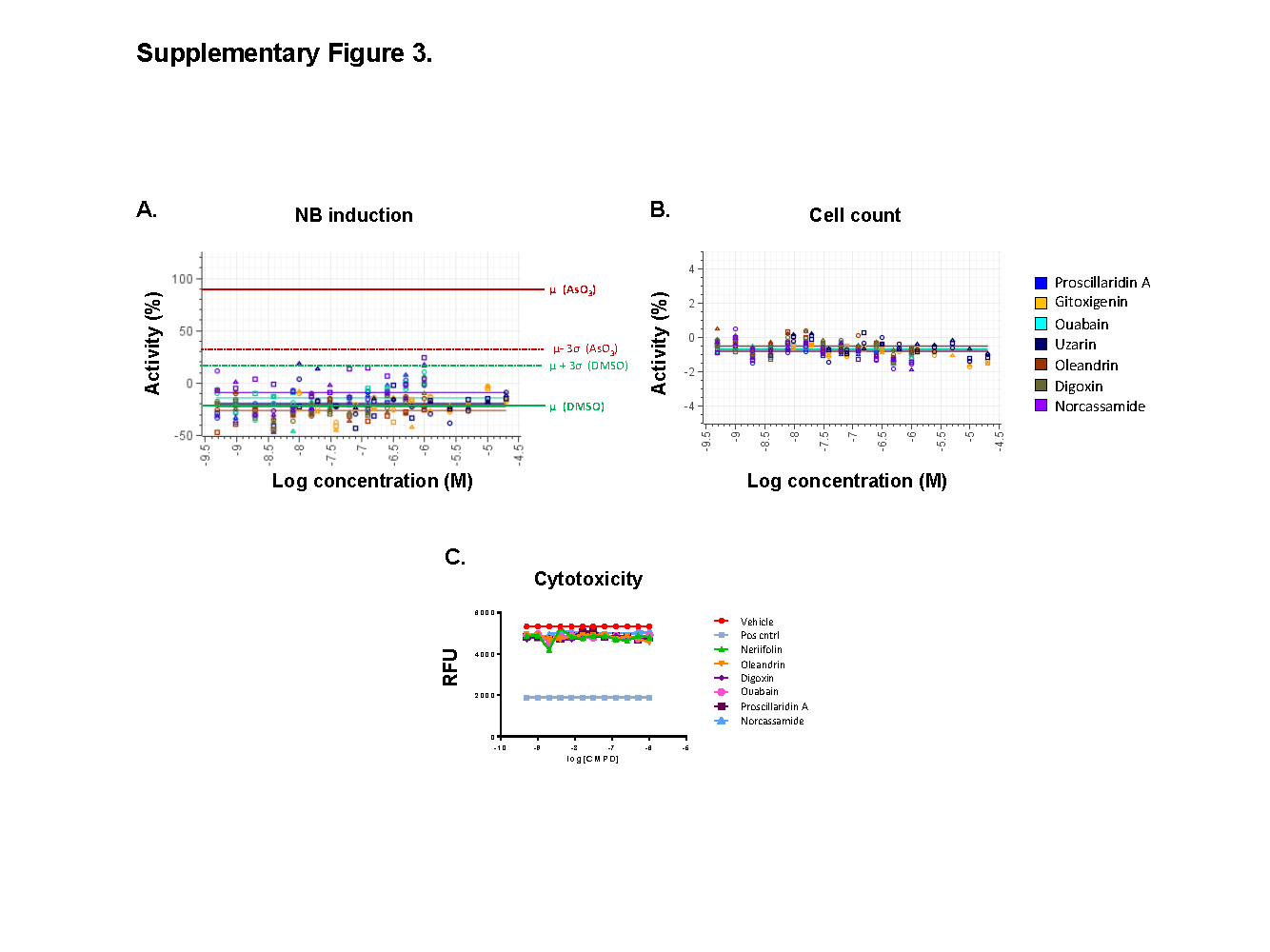

Supplement: S3 Fig — Murine IMDC-3 cells were treated with increasing concentrations of NKA inhibitors for 18h. PML NB formation (A), cell counts (B) and cytotoxicity (C) were determined as described in Material and Methods. Data are means of three replicates. (TIFF) [file pone.0152692.s004.tiff]

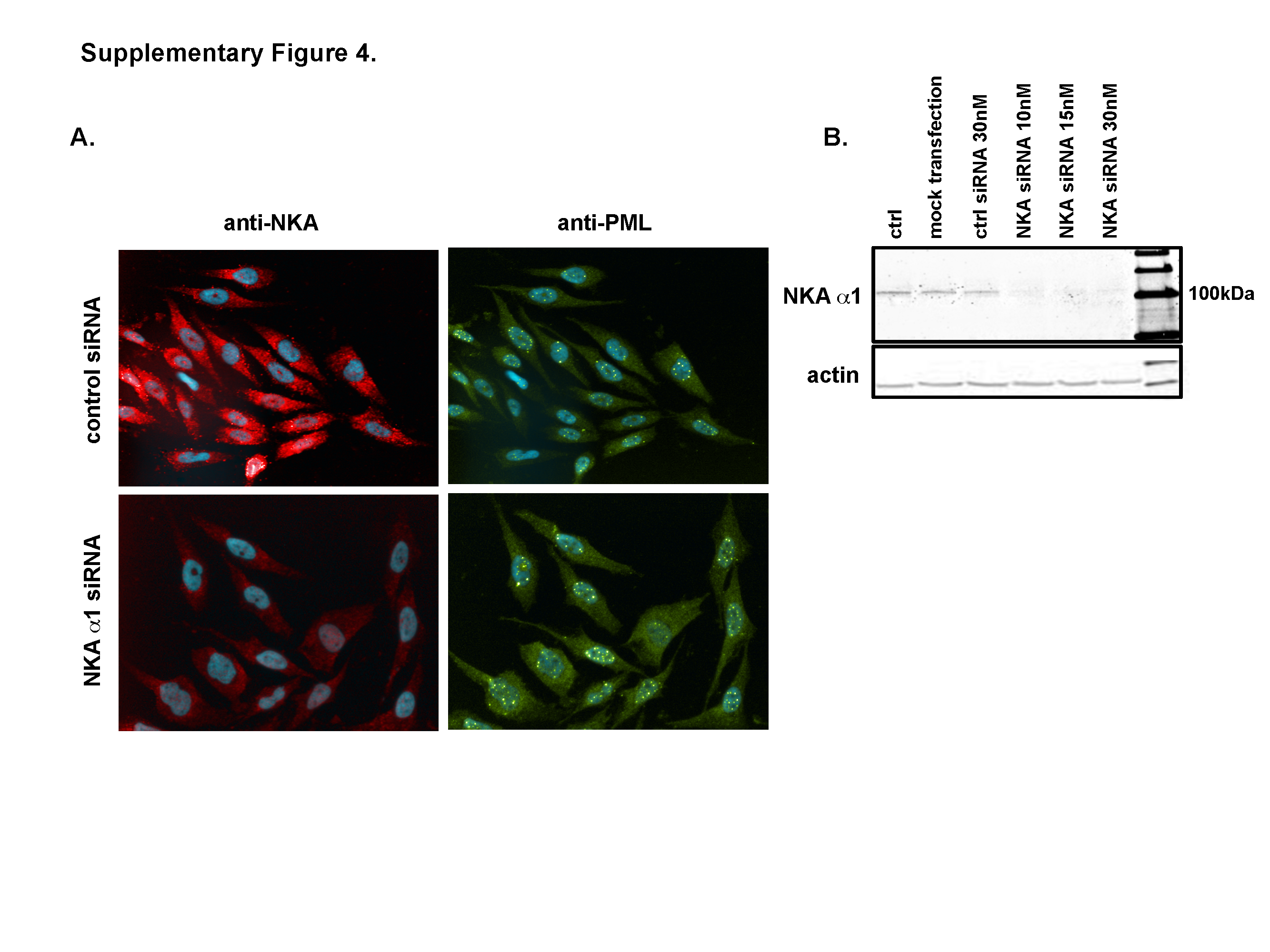

Supplement: S4 Fig — A, PPC-1 cells were seeded into 6 well plates at 200,000 cells/well. The next day the cells were transfected with 30 nM of control siRNA or 10, 15 and 30 nM of siRNA directed against human NKAα1. At 48h post transfection, the cells were fixed and stained with anti-PML antibody or DAPI. B, PPC-1 cells were cultured in 6-well plates at 200,000/well. The next day they were transfected as in A, and at 48h post-transfection the levels of NKAα1 and actin were determined by immunoblotting using anti-NKAα1 and anti-actin antibodies. (TIFF) [file pone.0152692.s005.tiff]

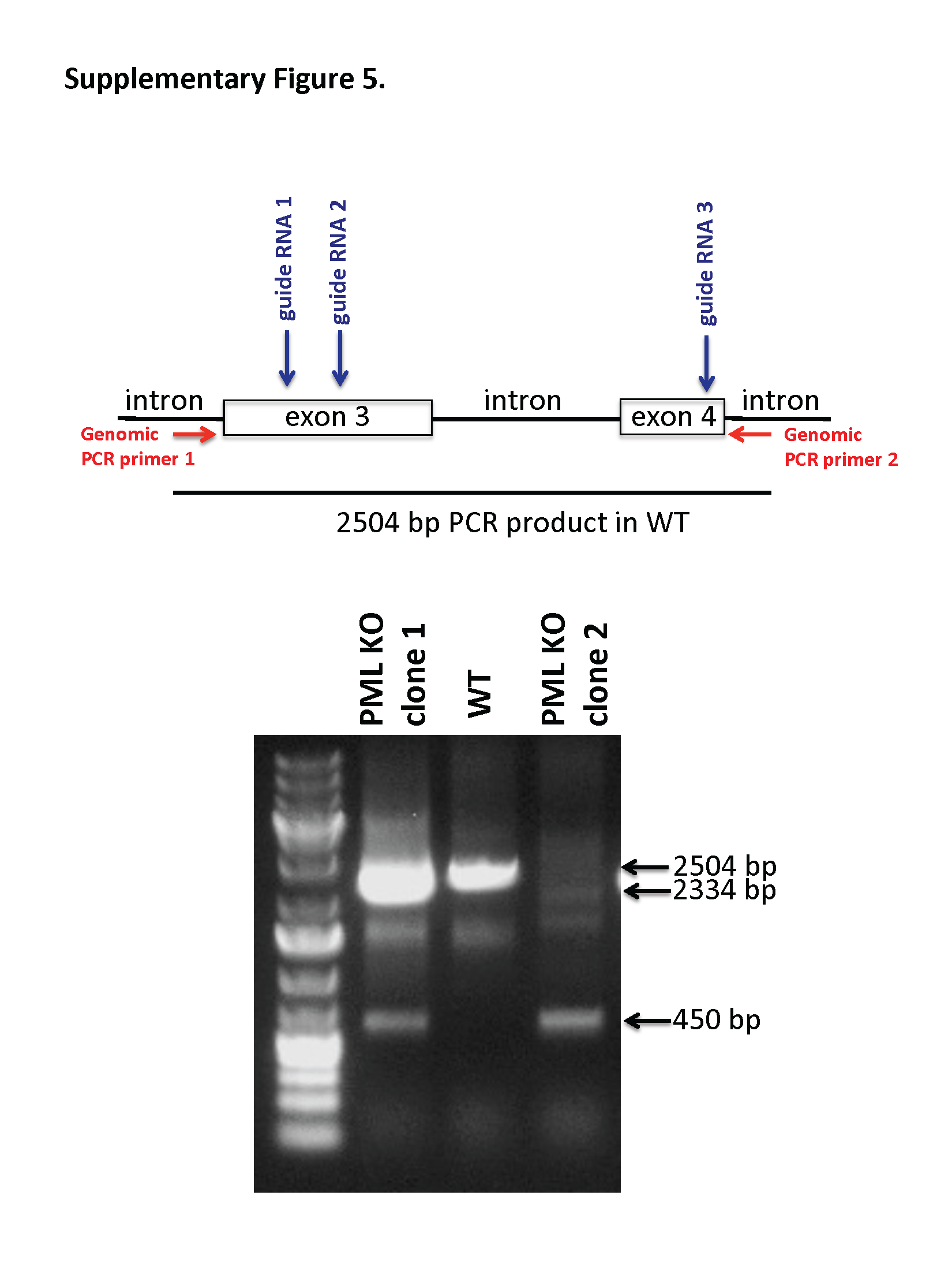

Supplement: S5 Fig — The scheme of PML genomic region, the sites targeted by the three guide RNAs and the genomic primers used to amplify 2504 bp region of the PML gene in the wild type (WT) HEK293T cells are shown. The lower panel shows the PCR amplification of the WT cells (2504bp), PML KO clone 1 (2334bp resulting from the excision between guide RNA 1 and guide RNA 2 and 450bp resulting from the excision between guide RNA 1 and guide RNA 3) and PML KO clone 2 (450bp resulting from the excision between guide RNA 1 and guide RNA 3). (TIFF) [file pone.0152692.s006.tiff]

S6 Fig.

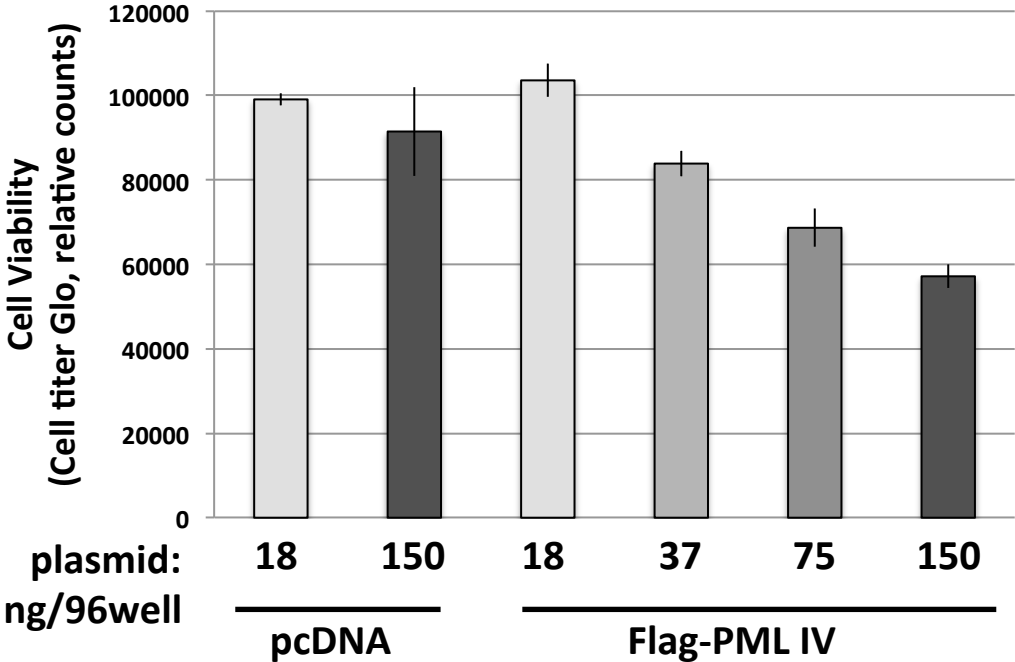

Supplement: S6 Fig — HEK293T cells were plated at a density of 5,000 cells/well in a 96 well plate. The cells were transfected with increasing amount of either empty vector pcDNA or Flag-PML IV (18-150ng/well). 48h after the transfection, the cell viability was assessed using Cell Titer Glo. Data are means of three replicates and the error bars are standard deviations. (PDF) [file pone.0152692.s007.pdf]

**S7 Fig.**

**No ouabain**

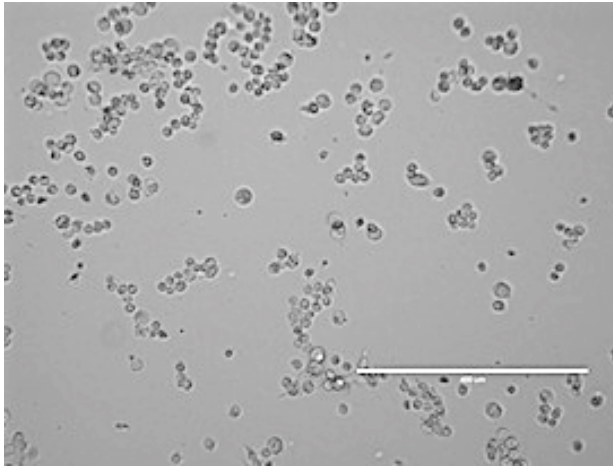

**25nM ouabain**

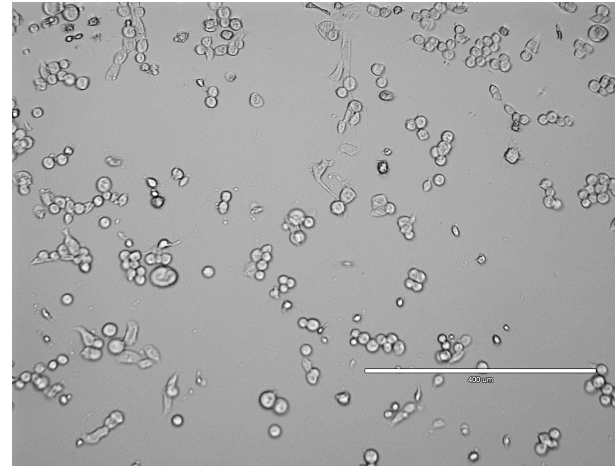

**50nM ouabain**

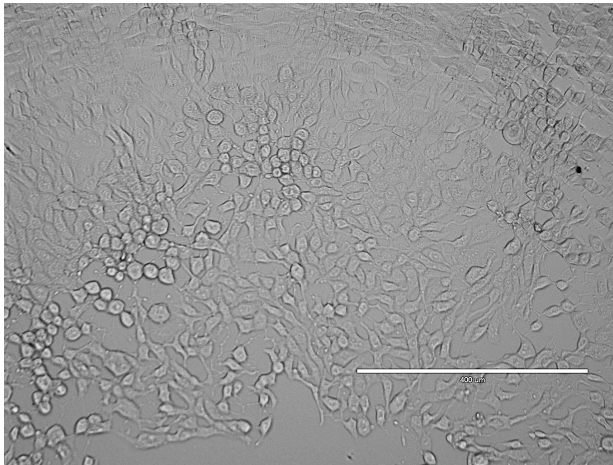

**100nM ouabain**

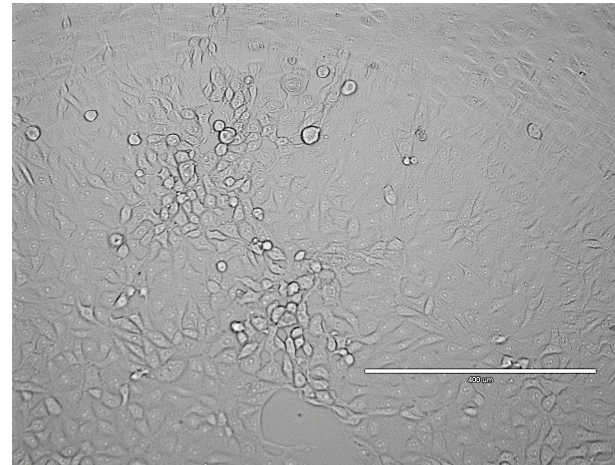

Supplement: S7 Fig — Vero cells were plated in Plate 1 at 90% confluency and were pre-treated with 0, 25, 50 or 100nM Ouabain for 5h, followed by infection with HSV-1 KOS for 24 hrs. The produced virus was harvested by 2 freeze-thaw cycles of media and cells in Plate 1. Then, one tenth of the produced virus was added to a new Plate 2 of Vero cells (70% confluent) and the cells were incubated for 48h before the pictures were taken (Note that Plate 2 was not treated with Ouabain). (PDF) [file pone.0152692.s008.pdf]
