# Supplementary material for: Cardiac Glycosides Activate the Tumor Suppressor and Viral Restriction Factor Promyelocytic Leukemia Protein (PML)
Source: PLoS One. 2016 Mar 31;11(3):e0152692. doi: 10.1371/journal.pone.0152692 (PMC4816303; doi:10.1371/journal.pone.0152692)
Supplement: S1 Table — The hits obtained in the primary screen were confirmed at two concentrations (10 μM for hit compounds based on PML activity, 1 μM for compounds showing significant cytotoxicity at 10 μM with increased PML activity in the remaining attached cells). Confirmed hits were tested in a funnel of secondary assays (phospho-H2AX staining, phospho-Chk1 staining, cytotoxicity dose-response, PML NB dose-response) to further eliminate artifacts. Additional cell line PPC-1 was also tested in cytotoxicity and PML NB assays. (DOCX) [file pone.0152692.s009.docx]

**S1 Table.** **Testing funnel of the hits obtained from the primary screen.**

| **Testing steps** | **Description** |
| --- | --- |
| Primary high throughput screen | 384-well PML NB HCS assay for 321,600 compounds at 10μM |
| Results from primary screen | 870 NB activators/cytotoxics |
| Hit confirmation assay | PML NB HCS assay at 1 μM and 10 μM |
| Confirmed hits | 76 Confirmed hits |
| Further testing | 73 Dry powders received |
| Secondary Assays | PML NB HCS dose response (HeLa, PPC1) |
|  | Cytotoxicity dose response (HeLa, PPC1) |
|  | p-H2AX, p-Chk1 dose response (HeLa) |
