# Supplementary material for: Cardiac Glycosides Activate the Tumor Suppressor and Viral Restriction Factor Promyelocytic Leukemia Protein (PML)
Source: PLoS One. 2016 Mar 31;11(3):e0152692. doi: 10.1371/journal.pone.0152692 (PMC4816303; doi:10.1371/journal.pone.0152692)
Supplement: S2 Table — EC50s for PML NB formation and cytotoxicity of various NKA inhibitors are determined following 18h treatment of HeLa and PPC-1 cells as described in Materials and Methods. The previously reported Kds for inhibition of NKAα1β1 by these compounds are also shown. NCI-60 means of GI50s are means of compound concentrations required to inhibit 50% of cell growth in a panel of 60 cancer cell lines as described in Materials and Methods. (DOCX) [file pone.0152692.s010.docx]

**S2 Table.** **Steroidal and non-steroidal NKA inhibitors’ activity.**

| **Compound Name** | **Human NKA Kd α1β1(nM)** | **NCI-60 mean**  **GI_50_ (nM)** | **HeLa**  **PML NB**  **EC_50_ (nM)** | **HeLa Cytotoxicity EC_50_ (nM)** | **PPC-1**  **PML NB**  **EC_50_ (nM)** | **PPC-1 Cytotoxicity EC_50_ (nM)** |
| --- | --- | --- | --- | --- | --- | --- |
| Proscillaridin A | 2.3 | 3.2 | 38 | 91 | 23 | 24 |
| Ouabain | 9.8 | 16 | 33 | 206 | 49 | 40 |
| Oleandrin | 35 | 10 | 107 | 176 | 48 | 50 |
| Digitoxin | 38 | 111 | 10 | 10 | 310 | 100 |
| Digoxin | 87 | 63 | 205 | 458 | 60 | 397 |
| Gitoxigenin | 500 | 1580 | 600 | 2,100 | 1,010 | 6,800 |
| Uzarin | 6,080 | 20,000 | 9,415 | 1061 | 10,425 | >20,000 |
| Hydroxynorerythrosuamide | ND | 400 | 250 | 2323 | 123 | 444 |
| Norcassamide | ND | 20 | 3.7 | 99 | 5.4 | 45 |
